# Cell-Type Specific Development of the Hyperpolarization-Activated Current, Ih, in Prefrontal Cortical Neurons

**DOI:** 10.3389/fnsyn.2018.00007

**Published:** 2018-05-11

**Authors:** Sha-Sha Yang, Yan-Chun Li, Austin A. Coley, Linda A. Chamberlin, Ping Yu, Wen-Jun Gao

**Affiliations:** ^1^Beijing Key Laboratory of Learning and Cognition, College of Psychology, Capital Normal University, Beijing, China; ^2^Department of Neurobiology and Anatomy, College of Medicine, Drexel University, Philadelphia, PA, United States

**Keywords:** pyramidal neurons, GABAergic interneurons, parvalbumin, HCN channel, H-current, prefrontal cortex, development, mouse

## Abstract

H-current, also known as hyperpolarization-activated current (Ih), is an inward current generated by the hyperpolarization-activated cyclic nucleotide-gated (HCN) cation channels. Ih plays an essential role in regulating neuronal properties, synaptic integration and plasticity, and synchronous activity in the brain. As these biological factors change across development, the brain undergoes varying levels of vulnerability to disorders like schizophrenia that disrupt prefrontal cortex (PFC)-dependent function. However, developmental changes in Ih in PFC neurons remains untested. Here, we examine Ih in pyramidal neurons vs. gamma-aminobutyric acid (GABA)ergic parvalbumin-expressing (PV+) interneurons in developing mouse PFC. Our findings show that the amplitudes of Ih in these cell types are identical during the juvenile period but differ at later time points. In pyramidal neurons, Ih amplitude significantly increases from juvenile to adolescence and follows a similar trend into adulthood. In contrast, the amplitude of Ih in PV+ interneurons decreases from juvenile to adolescence, and does not change from adolescence to adulthood. Moreover, the kinetics of HCN channels in pyramidal neurons is significantly slower than in PV+ interneurons, with a gradual decrease in pyramidal neurons and a gradual increase in PV+ cells across development. Our study reveals distinct developmental trajectories of Ih in pyramidal neurons and PV+ interneurons. The cell-type specific alteration of Ih during the critical period from juvenile to adolescence reflects the contribution of Ih to the maturation of the PFC and PFC-dependent function. These findings are essential for a better understanding of normal PFC function, and for elucidating Ih’s crucial role in the pathophysiology of neurodevelopmental disorders.

## Introduction

The prefrontal cortex (PFC) is extensively involved in cognitive and executive functions, and is a major area linked to the cognitive deficits observed in neurodevelopmental disorders such as depression, attention deficit hyperactivity disorder (ADHD) and schizophrenia (Diamond, 2011; Berridge et al., 2012; Monaco et al., 2015). A distinct feature of the PFC is its delayed maturation, especially involving the gamma-aminobutyric acid (GABA)ergic parvalbumin-expressing (PV+) interneurons (Lewis et al., 2005; Caballero and Tseng, 2016). Consequently, this causes the PFC to become vulnerable to any genetic and environmental factors that may lead to a disruption of normal development. Therefore, it is imperative to understand the physiological alterations in both PV+ interneurons and pyramidal neurons during stages of postnatal development.

H-current (Ih) is an inward current generated by the opening of hyperpolarization-activated cyclic nucleotide-gated (HCN) cation channels. Ih plays essential roles in regulating neuronal properties, synaptic integration and plasticity, and synchronous activity among neurons in the brain (Huang et al., 2011; He et al., 2014; Engel and Seutin, 2015; Gasselin et al., 2015; Masi et al., 2015). Ih also contributes to regulating the neuronal excitability and synaptic activities of both pyramidal neurons and GABAergic interneurons by maintaining the resting membrane potential (RMP) and after-hyperpolarization potential (AHP; Aponte et al., 2006; Bonin et al., 2013; Glykos et al., 2015). Ih has distinct regionally specific developmental patterns. In the mouse hippocampus, Ih increases several folds in CA1 and CA3 pyramidal neurons from postnatal day 1 (P1) to P20 (Vasilyev and Barish, 2002). In the substantia nigra, there is a remarkable increase in Ih amplitude from P1 to P18 (Washio et al., 1999). Additionally, in neonatal rat PFC, the cooperative activation of D1 and D2 receptors enhances Ih in layer I GABAergic interneurons (Wu and Hablitz, 2005). Although juvenile and adolescence are critical periods for PFC development and the progression of psychiatric disorders, the maturation process of Ih in prefrontal neurons during these critical developmental stages has not been characterized.

Here we examined the developmental change of Ih in pyramidal neurons vs. that of PV+ interneurons in the mouse PFC by using patch clamp recordings. We found that the Ih amplitude in both types of neurons was initially similar during the juvenile period, and later diverged in opposite directions. There was a significant amplitude increase in pyramidal neurons and a decrease in PV+ interneurons from juvenile to adolescence, resulting in several-fold higher amplitudes of Ih in pyramidal neurons vs. PV+ interneurons in adulthood. Our results indicate that Ih matures in a cell-type specific manner in prefrontal neurons.

## Materials and Methods

### Animals

Wild-type C57BL/6J mice (Jax stock # 000664, Bar Harbor, ME, USA), PV–Cre (+/−) mice (Jax stock # 008069), and Ai14-tdTomato (+/+) mice (Jax stock # 007914) were purchased from Jackson Laboratory (Bar Harbor, ME, USA). PV–Cre (+/−) mice and tdTomato (+/+) mice were used to breed PV–tdTomato animals in order to label PV–expressing interneurons with a td-Tomato marker. All animals were grouped as juveniles (P16–P21), adolescents (P35–P40) and adults (P60–P70) per our previous publications (Wang and Gao, 2009, 2010). The animals were maintained under standard housing conditions with food and water available *ad libitum* according to the National Institutes of Health guidelines. The protocol was approved by the Institutional Animal Care and Use Committee of Drexel University College of Medicine.

### Slices Preparation

Mice were anesthetized with Euthasol-III (0.2 ml/kg, Med-Pharmex Inc., Pomona, CA, USA) and decapitated. Brains were quickly removed and placed in ice-cold (<4°C) sucrose solution containing the following reagents (in mM): NaCl 87, sucrose 75, KCl 2.5, CaCl_2_ 1, MgCl_2_ 7, NaH_2_PO_4_ 1.25, NaHCO_3_ 25, glucose 25, which was aerated with 95% O_2_ and 5% CO_2_, pH 7.4. Horizontal cortical slices at 300 μm thickness were cut using Leica VT1200S (Leica Microsystems Inc., Buffalo Grove, IL, USA). Slices were then incubated for 40 min at 36°C before being maintained at room temperature until recording. Slices were submerged in a recording chamber filled with oxygenated artificial cerebrospinal fluid (ACSF, in mM): NaCl 124, KCl 2.5, CaCl_2_ 2, MgCL_2_ 1, NaH_2_PO_4_ 1.25, glucose 10, NaHCO_3_ 26, pH 7.4.

### *In Vitro* Electrophysiology

Whole-cell patch clamp recording was performed on layer V pyramidal neurons and PV+tdTomato-labeled interneurons in both prelimbic and infralimbic areas of the PFC. Pyramidal neurons were directly visualized and identified under an infra-red DIC via a video system installed in a Zeiss FS2 upright epifluorescent microscope under a 40× water-immersion lens. The PV+ interneurons were first visualized with the assistance of a fluorescent filter for tdTomato, and were further identified and recorded under DIC. Action potentials were recorded in current clamp mode with electrodes filled with potassium intracellular solution containing (in mM): K-gluconate 120, KCl 20, 4-(2-hydroxyethyl)-1-piperazineethanesulfonic acid (HEPES) 10, 0.1, Na_2_ATP 4, Na_2_GTP 0.3, Na_2_-phosphocreatine 5, osmolarity 304, pH 7.25 (adjusted with KOH). Ih was recorded in voltage clamp mode, with tetraethylammonium chloride (TEA, 30 mM, Sigma-Aldrich, St. Louis, MO, USA) added to the above intracellular solution to block delayed activated K+ current (Osmolarity: 314). Barium chloride (BaCl_2_ 1 mM, Sigma-Aldrich, St. Louis, MO, USA) was also added to the extracellular solution to block inward-rectifier K+ current in the Ih recording. Under voltage-clamp, the hyperpolarization voltage step command went from −60 mV to −130 mV by −5 mV increments, followed by a voltage step at −80 mV to monitor the capacitance of neurons. Under current-clamp, a series of 1500 ms hyperpolarizing currents (from −400 pA to 400 pA by 50 pA steps) was injected to test membrane response. Current-clamp recordings proceeded with neither TEA in the intracellular solution nor BaCl_2_ in the extracellular solution. RMP was directly measured in current-clamp mode after membrane breaking. Input resistance (IR) was calculated by a −100 pA hyperpolarizing current injection of 200 ms duration. The resistance of the recording glass pipette (Harvard Apparatus, Holliston, MA, USA) was 4–6 MΩ when measured with the intracellular solution. The series resistance during recording was 10–14 MΩ in current-clamp. The capacitance was also similarly auto-compensated. We monitored the series resistance change by checking the number before and after recording. Data were excluded when the change of series resistance exceeded 20%. We did not do any online- or offline-leak subtraction. All cells with significant leak were discarded without further recording. The signals were digitized at 10 kHz and low-pass filtered at 1 kHz. A selective Ih blocker ZD7288 (40 μM, Cayman Chemical Company, Ann Arbor, MI, USA) was acutely added to the extracellular solution to block HCN channels.

### Data Analysis

The acquired data were processed using the Clampfit 10 (Molecular Devices, Sunnyvale, CA, USA). Neurons with depolarized RMP (> −52 mV) were discarded without further analysis. Instantaneous current (I_Ins_), steady-state current (I_ss_), and current decay (tau) were calculated from the voltage-clamp recordings. Briefly, the I_Ins_ was located at the onset point of the 2-s hyperpolarizing step of each sweep and the I_ss_ was measured at the end of each 2-s hyperpolarizing step. The amplitude of Ih was measured by deducting I_Ins_ from I_ss_ at each sweep. Ih time constants were fitted with a standard exponential function of the equation: $f(t)=\sum_{i\;=\;1}^{n}Aie^{-t/\tau i}+\;C$ and expressed as the tau value in Clampfit. This is a basic function used to fit changes in current or voltage that are controlled by one or more first-order processes. The fit solves for the amplitude *A*, the time constant *τ*, and the constant *y*-offset *C* for each component *i*. The RMP, IR and AHP of pyramidal neurons and PV+ interneurons were measured from the current-clamp recordings. One-way ANOVA or two-way ANOVA analyses were performed on parameters by SPSS 23. For results with significant interaction effect in two-way ANOVA, simple effect test was conducted for further analysis. For results with insignificant interaction effect, Bonferroni’s *post hoc* test or Student *t*-test was further conducted as needed. All data are presented as mean ± standard error of the mean.

## Results

### Different Developmental Patterns of Ih in PFC Pyramidal Neurons and PV+ Interneurons

**Figure 1:**
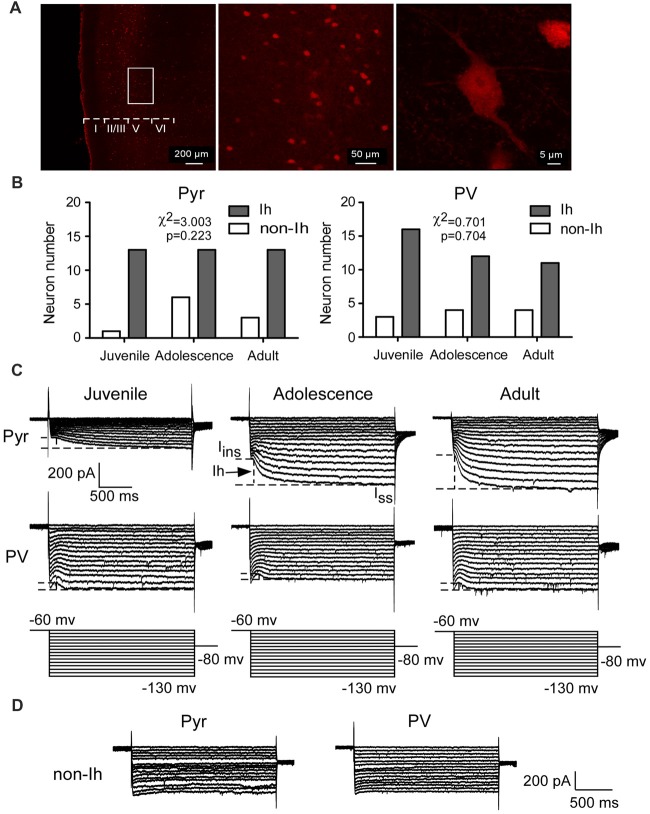
Hyperpolarization-activated current (Ih) is recorded from prefrontal pyramidal neurons and parvalbumin-expressing (PV+) interneurons during development. **(A)** Confocal images show the expression of PV+ interneurons labeled by td-Tomato in the prefrontal cortex (PFC). Scale bar = 200, 50 and 5 μm, respectively. **(B–D)** The numbers of pyramidal neurons and PV+ interneurons recorded from the layer 5 of the mPFC in each age group. PV+ interneurons were exclusively recorded from PV–tdTomato mice. Among these cells, a majority of the pyramidal neurons (39/49 or 78%) and the PV+ interneurons (39/50 or 80%) showed measurable Ih **(B,C)**, whereas the remaining cells exhibited no Ih **(D)**. **(C)** Representative current traces recorded from juvenile (P16–P21), adolescent (P35–P40) and adult (P60–P70) mice PFC pyramidal neurons and PV+ interneurons. The dash lines in the samples represent the measurements of Ih in every group. **(D)** Sample traces of neurons expressing no Ih.

To identify PV+ interneurons, we took confocal images of PFC slices from PV–tdTomato mice. As shown in Figure 1A, PV+ interneurons were successfully tagged with td-Tomato markers. Td-Tomato-labeled cells were evenly distributed across all layers from II–VI in the PFC. A total of 49 pyramidal neurons and 50 PV+ interneurons were used to test the properties of Ih from layer V of PFC across three age groups. Pyramidal neurons were collected from both wild-type mice and PV–tdTomato mice. We did not observe any difference in Ih amplitude or kinetics between the pyramidal neurons from these two mouse colonies, so the data were pooled together (Pyramidal cells from PV–tdTomato mice: −237 ± 40 pA, *n* = 7 vs. pyramidal neurons from C57BL6 mice: −208 ± 25 pA, *n* = 6; *t*_(11)_ = 0.58, *p* = 0.57). PV+ interneurons were recorded from PV–tdTomato mice. Among these cells, the majority of pyramidal neurons (39/49 or 78%) and PV+ interneurons (39/50 or 80%) showed measurable Ih (Figures 1B,C), whereas the remaining cells exhibited no detectible Ih (Figure 1D). More specifically, both Ih and non-Ih expressing cells among both pyramidal cells and PV+ interneurons showed similar distributions in different age groups (*χ*^2^ = 3.003, *p* = 0.223 for pyramidal interneuron (Pyr) and *χ*^2^ = 0.701, *p* = 0.704, Figure 1B).

**Figure 2:**
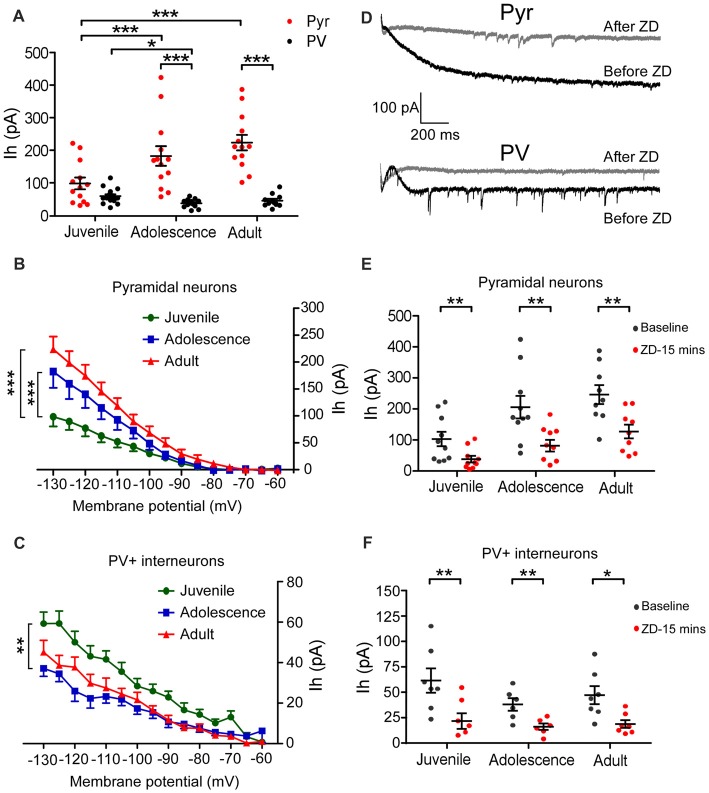
Pyramidal neurons and PV+ interneurons exhibit opposite age-dependent changes in Ih amplitude in the PFC. **(A)** Amplitudes of Ih measured at the end of the 2-s-long voltage step at −130 mV. There was a significant increase in Ih amplitude from juvenile to adolescence (*p* < 0.001) but no significant change from adolescence to adult (*p* > 0.05) in pyramidal neurons. In contrast, the changes in Ih amplitude in PV+ interneurons went an opposite direction compared to that of pyramidal neurons. During the juvenile period, there was no significant difference in Ih amplitude between pyramidal interneuron (Pyr) and PV+, but then Ih amplitude significantly decreased in PV+ interneurons from juvenile to adolescence (*p* < 0.05) but maintained the same level from adolescence to adult (*p* > 0.05). The data were analyzed with two-way ANOVA followed by a simple-effect test. **(B,C)** Relationship of voltage-current (Ih) in pyramidal neurons **(B)** and PV+ interneurons **(C)** in three age groups. One-way ANOVA is conducted at each step. The differences were gradually observed below −100 mV in both Pyr and PV+ cells from juvenile to adolescence. There were no differences between adolescence and adult groups in both Pyr and PV+ cells. **(D)** Example traces of Ih recorded at −130 mV before and after ZD7288 application. **(E,F)** Effect of ZD7288 (40 μM bath-application) on the amplitudes of Ih in prefrontal pyramidal neurons **(E)** and PV+ interneurons **(F)**. The changes in Ih amplitude were measured at 15 min after application of ZD7288 (paired *t*-test within each age group). ZD7288 had similarly significant blocking effects in all ages in both Pyr and PV+ neurons. *p < 0.05, **p < 0.01, ***p < 0.001.

In the presence of TEA and BaCl_2_, Ih was induced by the hyperpolarizing voltage steps (duration 2 s) from −60 mV to −130 mV by −5 mV increment. The amplitude of Ih was measured by subtracting the I_Ins_ from the I_ss_ at each step (Figure 1C). Two-way ANOVA was conducted with age × cell-type as independent variables. The differences between these two types of neurons were substantial. Pyramidal neurons possessed significantly larger Ih from adolescent to adult compared with that of PV+ interneurons (main effect of cell-type: *F*_(1,72)_ = 8.69, *p* < 0.001; Figure 2A). Due to the significant interaction effect (*F*_(2,72)_ = 4.99, *p* < 0.01), we performed the simple effect test. Specifically, the amplitude of Ih in pyramidal neurons significantly increased and almost doubled from juvenile to adolescence (juvenile: 98 ± 18 pA, adolescence: 182 ± 30 pA, *p* = 0.001), and slightly increased from adolescence to adulthood (adult: 224 ± 24 pA, *p* < 0.001 compared with juvenile and *p* > 0.05 compared with adolescence). In contrast, PV+ interneuronal Ih amplitude was comparable to that in pyramidal neurons during the juvenile period (*p* > 0.05). However, the Ih amplitude significantly decreased from juvenile to adolescence (juvenile: 59 ± 5.7 pA, adolescence: 37 ± 3.9 pA, *p* = 0.014) with a slight rebound in adulthood (adult: 45 ± 5.8 pA, *p* > 0.05 compared with both juvenile and adolescence; Figure 2A).

The current-voltage (I-V) relationship also showed significantly smaller Ih in pyramidal neurons from −100 mV step in juvenile animals compared with adolescence and adult (main effect of age: *F*_(2,540)_ = 40.41, *p* < 0.001; Figure 2B). In contrast, Ih in PV+ interneurons was significantly larger from −105 mV step in juvenile than in adolescence (main effect of age: *F*_(2,540)_ = 47.26, *p* < 0.001; Figure 2C).

We also confirmed the changes of Ih in 29 pyramidal neurons and 20 PV+ interneurons by bath-applying the HCN channel blocker ZD7288. ZD7288 had similar significant blocking effects in all age groups for both pyramidal neurons and PV+ interneurons. As ZD7288 requires 5–10 min to be fully effective, we analyzed the effects of ZD7288 15 min after bath-application to ensure the stability of the recording (cells that did not survive 15 min were excluded; Huang et al., 2017). As shown in Figures 2D–F, Ih was significantly blocked in pyramidal neurons 15 min after applying for ZD7288 (Paired *t*-test, juvenile: *n* = 10, *t*_(9)_ = 4.22, *p* = 0.002; adolescence: *n* = 10, *t*_(9)_ = 3.97, *p* = 0.003; adult: *n* = 9, *t*_(8)_ = 4.00, *p* = 0.004; Figure 2E) and PV+ interneurons (juvenile: *n* = 7, *t*_(6)_ = 6.35, *p* = 0.007; adolescence: *n* = 6, *t*_(5)_ = 4.13, *p* = 0.009; adult: *n* = 7, *t*_(6)_ = 3.50, *p* = 0.013; Figure 2F).

One potential concern regarding the recording was that Ih may not be fully isolated and thus the measurement could be contaminated by synaptic activity and other currents. We have therefore conducted additional experiments in the juvenile group to address this issue specifically, as in this age PV cells express the largest Ih. Glass pipettes were filled with the same intracellular solution as stated above including 30 mM TEA. After breaking the membrane, we first washed BaCl_2_ into the extracellular solution for 3–5 min. Ih was then recorded under voltage-clamp as described above. Then an extracellular solution containing following drugs were applied to fully isolate Ih: AMPA receptor antagonist 6,7-dinitroquinoxaline-2,3-dione (DNQX; 10 μM), N-methyl-D-aspartate (NMDA) receptor antagonist 3-(2-carboxypiperazin-4-yl)propyl-1-phosphonic acid (CPP; 10 μM), GABA_A_ receptor antagonist Picrotoxin (50 μM), and sodium channel blocker 3-(2-carboxypiperazin-4-yl)propyl-1-phosphonic acid (tetrodotoxin, TTX (0.5 μM). After 5 min wash in, Ih was recorded again. As shown in the Supplementary Figure S1, compared with the previous recording condition with BaCl_2_, the amplitudes of Ih recorded in both pyramidal neurons and PV+ interneurons under this additional condition were not significantly different.

Similar to our original results, Ih currents were significantly decreased in both pyramidal neurons and PV+ interneurons 15 min after 40 μM ZD7288 was added into the extracellular solution containing all drugs (Supplementary Figure S1B).

### Differential Kinetic Changes of HCN Channels in Pyramidal Neurons vs. PV+ Interneurons

**Figure 3:**
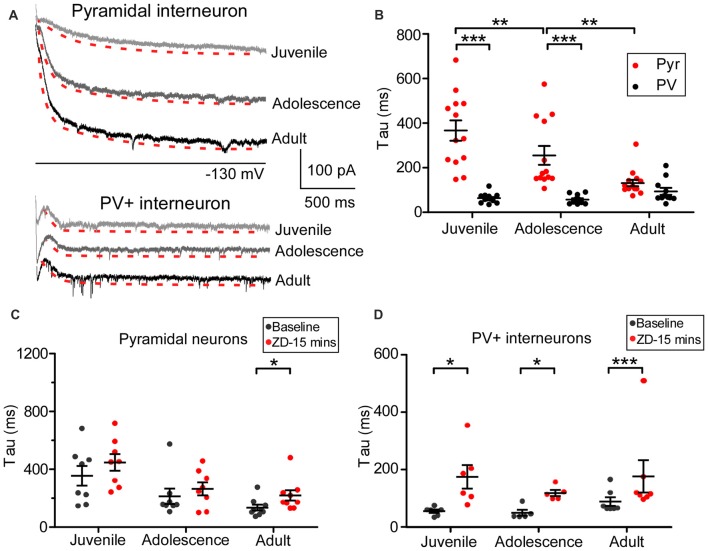
Cell type-specific development of hyperpolarization-activated cyclic nucleotide-gated (HCN) channel kinetics in the PFC. **(A)** Samples of the kinetics of Ih at −130 mV in PFC pyramidal neurons and PV+ interneurons. Traces of the tau measurement fit are shown in red dash lines, with a slightly lower shift for clarification in each case. **(B)** The developmental change in tau obtained from −130 mV in pyramidal neurons and PV+ interneurons in the PFC. Two-way ANOVA was performed with the simple-effect test. Ih in pyramidal neurons showed significantly slower decays in both juvenile and adolescence but not in adulthood compared with that of PV+ interneurons. **(C,D)** Effect of ZD7288 (40 μM) on HCN channel kinetics in prefrontal pyramidal neurons **(C)** and PV+ interneurons **(D)**. The change in tau was measured at 15 min after application of ZD7288 and was analyzed by paired *t*-test within each age group. Overall, ZD7288 did not have significant effects on the HCN channel kinetics in both juvenile and adolescence but did result in a significant increase in tau in adult pyramidal neurons. In contrast, ZD7288 significantly increased the decay of Ih in all three age groups in PV+ interneurons. *p < 0.05, **p < 0.01, ***p < 0.001.

The kinetics of Ih in pyramidal neurons is different from that of GABAergic interneurons in the adult hippocampus due to HCN channels isoforms (Yan et al., 2009; Dougherty et al., 2013; Omrani et al., 2015). As a result, we wondered if a similar difference exists among prefrontal cortical neurons, especially in regards to how the kinetics change from juvenile to adult periods. We plotted a standard exponential function to the recording traces and measured the tau value from the −130 mV step as an indicator of the HCN channel kinetics (showed in red in Figure 3A). Pyramidal neurons and PV+ interneurons exhibited different tau values in each of the three age groups. As shown in Figure 3B, the average tau of Ih in pyramidal neurons was significantly slower in the juvenile groups (367 ± 46 ms) than in the adolescent (255 ± 42 ms, simple effect test, *p* = 0.004 compared with juvenile) and in the adult groups (131 ± 14 ms, *p* < 0.001 compared with juvenile and *p* = 0.002 compared with adolescence). However, compared to pyramidal neurons, the tau for PV+ interneurons was significantly smaller (main effect of cell-type: *F*_(1,72)_ = 66.62, *p* < 0.001) with no significant change among the three age groups (juvenile: 64 ± 4.7 ms, adolescence: 57 ± 5.6 ms, adult: 94 ± 16 ms, simple effect test *p* > 0.05 among each two groups). These results suggest that the expression of HCN channel subunits with fast kinetics, most likely HCN1, increased during development in pyramidal neurons, while in the prefrontal PV+ interneurons, HCN2 may have increased during development. We also measured the effect of ZD7288 on tau in 25 pyramidal neurons and 17 PV+ interneurons. The application of ZD7288 induced different effects on these two types of PFC neurons during development. ZD7288 significantly reduced the tau of HCN channels in adult pyramidal neurons 15 min after application (*n* = 9, *t*_(8)_ = 3.19, *p* = 0.0129; Figure 3C). However, these effects were absent in juvenile and adolescent animals (juvenile: *n* = 8, *t*_(7)_ = 1.07, *p* = 0.321; adolescence: *n* = 8, *t*_(7)_ = 1.10, *p* = 0.309; Figure 3C). In the PV+ interneurons, ZD7288 significantly increased the tau in all age groups (juvenile: *n* = 6, *t*_(5)_ = 2.63, *p* = 0.047; adolescence: *n* = 5, *t*_(4)_ = 4.06, *p* = 0.015; adult: *n* = 6, *t*_(5)_ = 11.47, *p* < 0.001; Figure 3D). These results suggest that ZD7288 may have high affinity to HCN1 subunit. Lack of HCN1 in immature pyramidal neurons result in the unchanged of tau by ZD7288.

### HCN Channel Blockade Alters the Passive Membrane Properties of Pyramidal Neurons in PFC

**Figure 4:**
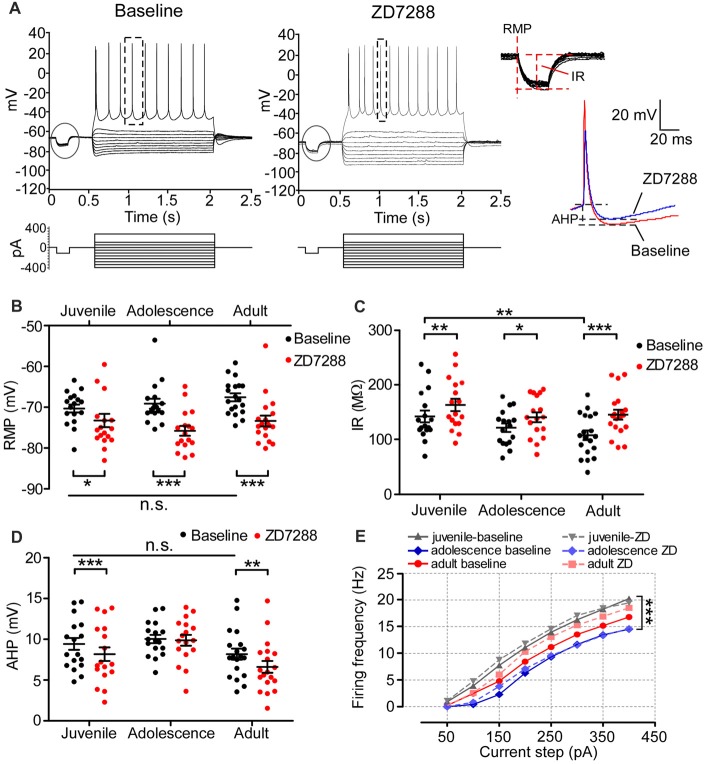
The contribution of Ih on the passive membrane properties and excitability of pyramidal neurons in the PFC. **(A)** Typical responses of membrane potential of pyramidal neurons to a series of current injection in current-clamp before (left) and after (middle) applying ZD7288. The steps enclosed in the circle were magnified to demonstrate the measurement of resting membrane potentials (RMP) and input resistance (IR). Samples of action potential spikes from a pyramidal neuron (dashed lines, right panel) show the measurements of after-hyperpolarization potential (AHP). **(B–D)** Changes in RMP **(B)**, IR **(C)** and AHP **(D)** induced by application of ZD7288 (40 μM) in different age groups. Two-way ANOVA was conducted with *post hoc* test. Blocking Ih exhibited strong significant effects on RMP and IR in juvenile, adolescent and adult groups. However, for AHP, ZD7288 had significant effects in juvenile and adulthood but not in adolescence. **(E)** The excitability of pyramidal neurons was not significantly altered by blocking Ih, measured as firing frequency in response to the depolarizing current step (duration 1.5 ms). *p < 0.05, **p < 0.01, ***p < 0.001. Note: the error bars in **(E)** were omitted for clarity.

A previous study demonstrated that HCN1 in adult PFC pyramidal neurons is critical for maintaining passive membrane properties (Thuault et al., 2013). However, how Ih affects the neural membrane properties of prefrontal neurons during development remains uncharacterized. As shown in the recording samples in Figure 4A, we injected a series of current into 54 pyramidal neurons to test the membrane response, including RMP and IR, in all age groups. The typical action potential (usually the fourth spike from the first sweep with APs) chosen to measure AHP is displayed in the right panel of Figure 4A. Neither the RMPs (one-way ANOVA, *F*_(2, 50)_ = 1.50, *p* = 0.234; Figure 4B) nor AHPs (*F*_(2,50)_ = 2.06, *p* = 0.138; Figure 4D) of pyramidal neurons changed during development. The IR in adult was significantly lower than in juvenile (*F*_(2,50)_ = 3.52, *p* = 0.037; *post hoc* test, juvenile vs. adult, *t* = 2.65, *p* < 0.01), but not lower than adolescence (juvenile vs. adolescence, *t* = 1.55, *p* > 0.05; adolescence vs. adult, *t* = 1.06, *p* > 0.05; Figure 4C). We then bath applied ZD7288 to test the changes in RMP, IR and AHP after blocking Ih in each age group. Our results indicated that after blocking Ih in pyramidal neurons, the RMP was significantly hyperpolarized in all age groups, including juvenile (baseline: −70 ± 0.97 mV, ZD7288: −74 ± 1.6 mV, *t*_(16)_ = 2.57, *p* = 0.021), adolescence (baseline: −69 ± 1.2 mV, ZD7288: −76 ± 1.2 mV, *t*_(16)_ = 7.39, *p* < 0.001) and adult (baseline: −68 ± 0.97 mV, ZD7288: −73 ± 1.3 mV, *t*_(18)_ = 4.94, *p* < 0.001; Figure 4B). Correspondingly, the IR was also significantly increased in juvenile (baseline: 142 ± 11 MΩ, ZD7288: 163 ± 11 MΩ, *t*_(16)_ = 3.54, *p* = 0.003;) adolescence (baseline: 122 ± 7.8 MΩ, ZD7288: 141 ± 9.0 MΩ, *t*_(16)_ = 2.19, *p* = 0.044) and adults groups (baseline: 108 ± 8.8 MΩ, ZD7288: 145 ± 9.0 MΩ, *t*_(18)_ = 4.48, *p* < 0.001; Figure 4C) after blocking HCN channels. The response of AHP to ZD7288 was different from that of RMP or IR. The application of ZD7288 induced a significant decrease in AHP in juvenile (baseline: 9.3 ± 0.75 mV, ZD7288: 8.2 ± 0.83 mV, *post hoc*, *t*_(16)_ = 4.04, *p* = 0.001) and adult pyramidal neurons (baseline: 8.2 ± 0.68 mV, ZD7288: 6.6 ± 0.72 mV, *t*_(16)_ = 3.01, *p* = 0.008), but not in adolescent ones (baseline: 10 ± 0.51 mV, ZD7288: 9.9 ± 0.66 mV, *t*_(18)_ = 0.32, *p* = 0.75; Figure 4D). Although the passive membrane properties were altered in response to the blocking of Ih, the excitability of pyramidal neurons was not influenced by ZD7288 in any age group. The spike frequency in juvenile cells was significantly higher than in adolescent cells (*p* < 0.001) with adult cells in between (*p* > 0.05 with both juvenile and adolescence; Figure 4E). There were no significant changes in action potential frequency in all three age groups after applying ZD7288 in PFC pyramidal neurons (*p* > 0.05 for all, Figure 4E).

### Blocking HCN Channels Alters the Passive Membrane Properties of PV+ Interneurons in PFC

**Figure 5:**
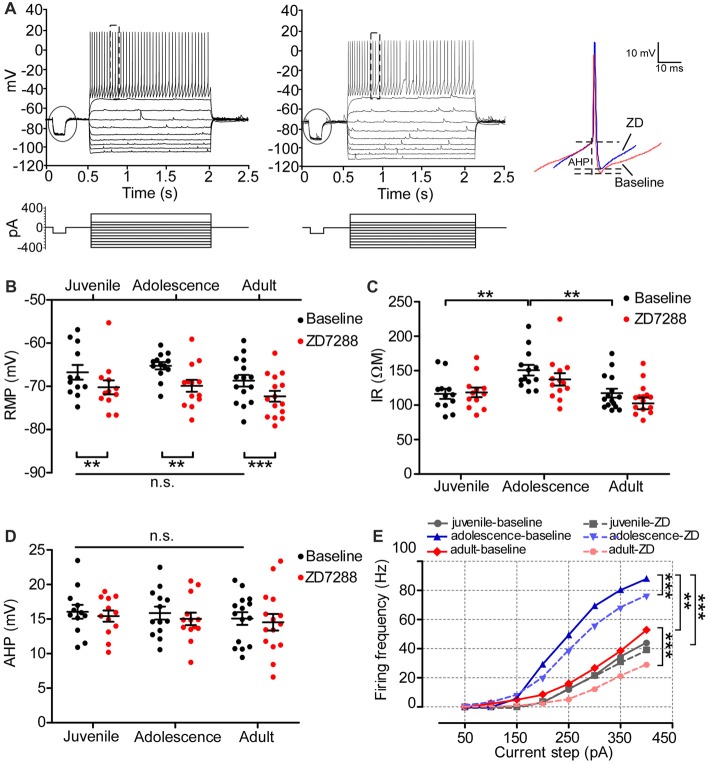
The contribution of Ih on the passive membrane properties and excitability of PV+ interneurons in the PFC. **(A)** Typical responses of membrane potential of a PV+ interneuron to a series of current injection in current-clamp before (left) and after (middle) applying ZD. The steps enclosed in the circle were used for measurements of RMP and IR as exhibited in Figure 4. Samples of action potential spikes from a PV+ interneuron (dashed lines, right panel) show the measurement of AHP. **(B–D)** Changes in RMP **(B)**, IR **(C)** and AHP **(D)** induced by application of ZD7288 (40 μM) in different age groups. Two-way ANOVA was conducted with *post hoc* test. PV+ neurons showed significantly higher IR during adolescence but no changes in RMP or AHP. Blocking Ih had no effect on IR or AHP, but significantly hyperpolarized PV+ interneurons by decreasing the RMP in all age groups. **(E)** The excitability of PV+ interneurons, measured as firing frequency in response to the depolarizing current step (duration 1.5 ms), was not significantly altered by blocking Ih in juvenile, but were significantly decreased in adolescence and adult. **p < 0.01, ***p < 0.001. Note: the error bars in **(E)** were omitted for clarity.

The properties and function of Ih in PFC GABAergic interneurons have not been adequately addressed. A previous study reported an enhancement of Ih by a cooperative activation of DA D1 and D2 receptors in layer I interneurons in the neonatal rat PFC (Wu and Hablitz, 2005). This led us to wonder how Ih maintains the passive membrane properties and neural excitability of PFC PV+ interneurons during development. We measured the change in RMP, IR and AHP before and after ZD7288 in 40 PV+ interneurons in the developing PFC. The sample recording displayed in Figure 5A represents a typical fast-spiking (FS) PV+ interneuron. We found that the maturation of the PFC did not influence RMP or AHP of PV+ interneurons. There was no significant change in RMP (one-way ANOVA, *F*_(2,37)_ = 1.77, *p* = 0.184; Figure 5B) or AHP (*F*_(2,37)_ = 0.31, *p* = 0.737; Figure 5D) among the three age groups. However, IR was significantly larger in adolescent PV+ interneurons (baseline: 151 ± 7.8 MΩ) compared with that of juvenile PV+ interneurons (baseline: 116 ± 7.3 MΩ, one-way ANOVA, *F*_(2,37)_ = 7.32, *p* = 0.002, *post hoc*, *t* = 3.28, *p* < 0.01) and of adult (baseline: 117 ± 6.5 MΩ, *t* = 3.36, *p* < 0.01; Figure 5C), exhibiting an inverted-U development pattern. Similarly, we tested the role of Ih in maintaining the membrane properties of PV+ interneurons. The application of ZD7288 induced a significant hyperpolarization with lower RMP in PV+ interneurons in all three age groups (juvenile-baseline: −67 ± 1.7 mV, ZD7288: −70 ± 1.6 mV, *t*_(11)_ = 3.17, *p* = 0.009; adolescence-baseline: −65 ± 0.85 mV, ZD7288: −70 ± 1.4 mV, *t*_(12)_ = 4.26, *p* = 0.001; adult-baseline: −69 ± 1.4 mV, ZD7288: −72 ± 1.3 mV, *t*_(14)_ = 5.62, *p* < 0.001; Figure 5B). In contrast, the IR (ZD7288: juvenile, 119 ± 6.9 MΩ, *t*_(11)_ = 0.50; adolescence, 137 ± 8.9 MΩ, *t*_(12)_ = 2.15; adult, 103 ± 8.5 MΩ, *t*_(14)_ = 1.39, *p* > 0.05 for all) and AHP (juvenile: baseline, 16 ± 0.99 mV, ZD7288, 15 ± 0.82 mV, *t*_(11)_ = 0.87; adolescence: baseline, 16 ± 0.95 mV, ZD7288, 15 ± 0.91 mV, *t*_(12)_ = 1.57; adult: baseline, 15 ± 0.92 mV, ZD7288, 15 ± 1.2 mV, *t*_(14)_ = 0.85, *p* > 0.05 for all) were not affected by blocking the HCN channels in any age group (Figures 5C,D). We also examined the effects of Ih on the excitability of PV+ interneurons by comparing the action potential frequency among all age groups. The action potential displayed distinct age-dependent development. In particular, adolescent PV+ interneurons displayed significantly higher firing frequency compared with juvenile (*p* < 0.001) and adult (*p* < 0.01; Figure 5E), and again, exhibited an inverted-U developmental change with adolescent PV+ interneurons showing higher excitability. Blocking Ih failed to alter the frequency in PV+ interneurons in the juvenile period (*p* > 0.05) but significantly decreased the firing frequency in adolescence (*p* < 0.001) and adulthood (*p* < 0.001; Figure 5E).

## Discussion

Previous studies have revealed a considerable change in HCN channels from early life to adulthood in the central nervous system (Vasilyev and Barish, 2002; Kanyshkova et al., 2009; Cho et al., 2011; Seo et al., 2015). However, the development of Ih in the PFC, especially the difference between pyramidal neurons and PV+ interneurons, remains untested. To our knowledge, our study is the first to examine the development of Ih in both types of neurons in the mouse PFC. We discovered that in prefrontal cortical neurons, Ih develops in a distinct cell-type specific manner. Specifically, we found that the amplitude of Ih in pyramidal neurons vs. that in PV+ interneurons was initially similar during the juvenile period, but eventually diverged in opposite directions. Ih amplitude in pyramidal neurons significantly increased from juvenile to adolescence but exhibited no significant change from adolescence to adulthood. In contrast, the amplitude of Ih in PV+ cells decreased from juvenile to adolescence with a non-significant rebound in the adult. Furthermore, the kinetics of HCN channels in pyramidal neurons was significantly slower than in PV+ interneurons, with a gradual decrease in pyramidal neurons in contrast to a gradual increase in PV+ cells during development. Ih also contributed to maintaining the RMP, IR and AHP in pyramidal neurons and RMP PV+ interneurons.

### Opposite Pattern of Ih Development in Pyramidal Neurons vs. That of PV+ Interneurons in the PFC

The development of Ih reflects a change of HCN channels. Our results reveal a significant increase in Ih amplitude in pyramidal neurons during postnatal development in PFC, suggesting an increase in HCN channels. This finding is similar to previous reports on the hippocampal pyramidal neurons and thalamic relay neurons, but a regional difference is clear. In hippocampal pyramidal neurons, Ih amplitude increases dramatically in early development from P1 to P20 (Vasilyev and Barish, 2002), whereas in thalamic relay neurons, Ih gradually increases from P1 to P90 (Kanyshkova et al., 2009). In contrast, Ih amplitude in the pyramidal neurons of the PFC exhibits a unique age-dependent alteration, with a significant increase from juvenile to adolescence, reflecting the critical developmental stage of the PFC. Meanwhile, the tau of Ih in pyramidal neurons displayed a gradual decrease from juvenile to adult. HCN1 is the subunit with fastest kinetic among HCN channel family. Therefore, we speculate that increase expression of HCN1 subunit contributes the gradual increase of tau in pyramidal neurons during development. An increase in HCN1 and HCN2 is reported in the hippocampus and subcortical neurons throughout postnatal development (Bender et al., 2001; Kanyshkova et al., 2009; Seo et al., 2015) but remains uncharacterized in the PFC. Previous studies reported a majority of HCN1 and HCN2 subunits in the adult neocortex (Bender and Baram, 2008; Cho et al., 2011), but whether this is also the case for PFC neurons remains to be determined by further study.

In contrast to the increase in Ih amplitude across development seen in pyramidal neurons, the amplitude of Ih in PV+ interneurons started similarly to that in pyramidal neurons during the juvenile period but significantly decreased in adolescence. There was a slight rebound from adolescence to adulthood, exhibiting a small U-shaped development. However, the tau did not show a similar change, suggesting that the Ih in PV+ interneurons may decrease in early development without a subunit shift in the developing PFC. We also noticed a significant small tau for HCN channels in PV+ interneurons, suggesting that the native channel in PV+ interneurons is most likely dominated by the HCN1 monomer in immature groups, as the tau value in PV interneurons is quite close to what previous reported about HCN1 kinetics (Thuault et al., 2013). The slight increase of tau in adult PV+ interneurons suggested increase of slow HCN subunits in adult group. This is different from the previous study of HCN channels in the hippocampus, where equally high levels of HCN1 and HCN2 were detected in PV+ basket cells from the juvenile dentate gyrus (Aponte et al., 2006). In the adult hippocampus, higher levels of immunopositive HCN4 in both CA1 and dentate gyrus PV+ interneurons were observed (Hughes et al., 2013). Therefore, the isoform distribution of HCN channels is highly dependent on neural sub-population (Bender et al., 2001; Leist et al., 2016). Further studies are necessary to identify the isoform of HCN channels in different types of GABAergic interneurons in the PFC.

### Ih Differentially Modulates the Membrane Properties of Pyramidal Neurons and PV+ Interneurons

The function of Ih is demonstrated to be cell-type specific. Previous studies have suggested that pyramidal neurons preferentially express HCN channels on dendrites and cell bodies, while in GABAergic interneurons, HCN channels are mainly located on the axon terminals to mediate presynaptic GABA release (Biel et al., 2009; Cho et al., 2011; Huang et al., 2011; Han et al., 2017). The location of these channels on the neuron consequently plays different roles in controlling passive membrane properties and postsynaptic integration. We found that blocking HCN channels similarly and significantly reduced RMP of pyramidal neurons in all age groups from juvenile to adult. Correspondingly, IRs of pyramidal neurons were also significantly increased by ZD7288 in all three age groups. The reduction of AHP in juvenile and adult pyramidal neurons in our study is consistent with findings from a previous study (Bonin et al., 2013). We did not observe an effect on the frequency of action potentials in pyramidal neurons after blockade of Ih. One possible explanation is that an increased post-synaptic summation resulting from the blockade of HCN channels on dendrites compensated for the hyperpolarization of RMP induced by blockade of Ih on soma (Engel and Seutin, 2015). This, however, requires further experimental evidence.

We have also noted that although the amplitude of Ih in PV+ interneurons is relatively small and remains stable without dramatic changes during the development, it exerts a more powerful effect on the RMP and neuronal excitability compared to that of pyramidal neurons. Our results show that blocking HCN channels significantly hyperpolarizes the RMP of PV+ interneurons in all age groups. Blocking Ih also causes a remarkable frequency decrease of action potential firing in adolescent and adult PV+ interneurons, but has no effect on pyramidal neuron firing. Furthermore, Ih in PV+ interneurons has fast kinetics, a property of HCN1 channels. HCN1 is usually activated by significantly lower hyperpolarized voltages compared to other isoforms (Altomare et al., 2003; Baruscotti et al., 2005). These properties enable the small Ih in PV+ interneurons to have a strong influence on RMP. Interestingly, blocking HCN1 does not change the IR and AHP in PV+ interneurons, and the reason for this discrepancy remains to be determined.

### Ih Contributes to the Functional Development of PFC in a Cell-Type Specific Manner

The most important finding in our study is the opposite development pattern of Ih in pyramidal neurons and PV+ interneurons. This distinct cell-type specific development of Ih raises additional questions concerning on how HCN channel changes in pyramidal neurons and PV+ interneurons would differentially affect normal PFC development and the pathophysiological process of neurodevelopmental disorders (He et al., 2014). For example, how does the maturation process of Ih contribute to the functional development of the PFC? Would disrupting the normal development of Ih in either pyramidal neurons or PV+ interneurons cause synaptic, cellular, and behavioral dysfunction of the PFC? In addition, how is this disruption associated with the pathological processes of diseases such as schizophrenia, autism, depression, and ADHD? Our data suggest that the increase in Ih amplitude in prefrontal pyramidal neurons from juvenile to adolescence is highly dependent on the augmentation of HCN1 density. Disrupting the dramatic increase of Ih in pyramidal neurons during these critical periods would provide a pathophysiological basis for PFC-dependent cognitive dysfunction in adulthood. Indeed, completely knocking out HCN1 from PFC pyramidal neurons results in a working memory deficit (Thuault et al., 2013). We recently also reported that treatment with methylphenidate produced significant depressive effects on pyramidal neurons by increasing Ih in the juvenile rat PFC, while exerting excitatory effects in adult rats (Urban et al., 2012). In addition, a recent study found that SHANK3 mutations severely and specifically impaired Ih channels, and that mouse SHANK3-deficient neurons exhibited a dramatic decreased Ih currents, suggesting the importance of HCN channels in Ih channelopathy, and the potential for pharmacological intervention targeting Ih (Yi et al., 2016).

In summary, HCN channels are critical for maintaining intrinsic membrane properties and neuron excitability. However, the role of HCN channels in mental disorders with cognitive symptoms, such as schizophrenia, has yet to be elucidated; how Ih is altered in developing prefrontal neurons has not been tested. Our study reveals distinct developmental trajectories of Ih in pyramidal neurons and PV+ interneurons. The cell-type specific alteration of Ih during postnatal development, especially during the critical period from juvenile to adolescence, reflects the specific function and unique contribution of Ih in two types of neurons for maturation of the PFC and PFC-dependent function. This study is the first attempt to determine a cell-type specific development of Ih in PFC pyramidal neurons compared to that of PV+ interneurons. It is also the first study selectively investigating Ih properties in PV+ interneurons at different ages from juvenile to adulthood. These findings are not only essential for better understanding of normal PFC function but also for elucidating Ih’s crucial role in the pathophysiological processes of neurodevelopmental disorders.

## Author Contributions

S-SY and W-JG conceived and designed the study. S-SY performed nearly all experiments and analyzed data. Y-CL instructed the *in vitro* electrophysiological recording and data analysis methods. AAC and LAC performed some of the experiments. S-SY performed the confocal imaging and wrote the manuscript with feedback from all authors. W-JG and PY supervised the study.

## Conflict of Interest Statement

The authors declare that the research was conducted in the absence of any commercial or financial relationships that could be construed as a potential conflict of interest.

## Abbreviations

ACSF: artificial cerebrospinal fluid
ADHD: attention deficit hyperactivity disorder
AHP: after-hyperpolarization potential
BaCl_2_: barium chloride
cAMP: cyclic adenosine monophosphate
CPP: 3-(2-carboxypiperazin-4-yl)propyl-1-phosphonic acid
DA: dopamine
DNQX: 6,7-dinitroquinoxaline-2,3-dione
EGTA: ethylene glycol-bis(β-aminoethyl ether)-N,N,N′,N′-tetraacetic acid
FS: fast-spiking
GABA: gamma-aminobutyric acid
HCN channels: hyperpolarization-activated cyclic nucleotide-gated cation channels
HEPES: 4-(2-hydroxyethyl)-1-piperazineethanesulfonic acid
Ih: hyperpolarization-activated current
IR: input resistance
NMDA: N-methyl-D-aspartate
P#: postnatal day
PFC: prefrontal cortex
PV+: parvalbumin-expressing
pyr: pyramidal interneuron
RMP: resting membrane potential
TEA: tetraethylammonium chloride
TTX: tetrodotoxin
ZD: ZD7288.
