# Supplementary material for: Cell-Type Specific Development of the Hyperpolarization-Activated Current, Ih, in Prefrontal Cortical Neurons
Source: Front Synaptic Neurosci. 2018 May 11;10:7. doi: 10.3389/fnsyn.2018.00007 (PMC5958189; doi:10.3389/fnsyn.2018.00007)
Supplement: Supplementary file 1 [file Data_Sheet_1.docx]

***Yang et al Supplemental Materials***





Figure S1. Ih currents recorded with blockade of synaptic activity and sodium channel are comparable with those recorded in intracellular solution containing only TEA+BaCl_2_. A, pyramidal neurons: TEA+BaCl_2_, 118±27 pA vs all drugs, 112±25 pA; Tukey’s post-hoc test, q=0.55, p>0.05, n=9; ZD7288, 50±19 pA; F_(2,8)_=11.15, p<0.001, Tukey’s post-hoc, BaCl_2_ vs ZD7288, q=6.04, p<0.01, all drugs vs ZD7288, q=5.49, p<0.01. B, PV+ interneurons: TEA+BaCl_2_, 61±9.0 pA vs all drugs, 55±8.7 pA; Tukey’s post-hoc test, q=1.23, p>0.05, n=8. ZD7288, 21±3.1pA; F_(2,7)_=24.68, p<0.001, Tukey’s post-hoc test, BaCl_2_ vs ZD7288, q=9.15, p<0.001, all drugs vs ZD7288, q=7.92, p<0.001. ** p<0.01, *** p<0.001.
